# A New Biocontrol Tool to Fight Potato Late Blight Based on *Willaertia magna* C2c Maky Lysate

**DOI:** 10.3390/plants11202756

**Published:** 2022-10-18

**Authors:** Sandrine Troussieux, Annabelle Gilgen, Jean-Luc Souche

**Affiliations:** R&D Department, Amoéba, 69680 Chassieu, France

**Keywords:** *Phytophtora infestans*, biofungicide, amoeba

## Abstract

Potato late blight (PLB) is one of the most destructive disease affecting potatoes. Late blight control relies almost exclusively on the use of chemical pesticides, including copper products, which are efficient but controversial due to their environmental toxicity. Societal pressure and the quest for more sustainable agriculture reinforce the need for natural plant protection products. To respond to this demand, we tested the lysate of the amoeba *Willaertia magna* C2c Maky on PLB. This active substance exhibits plant protection properties against grape downy mildew thanks to a dual mode of action (plant elicitor and antifungal direct effect). We hypothesized that this active substance might also have an effect against other diseases caused by oomycetes on other crops, such as potato. In vitro, in planta, and in-field studies were conducted. The collected data demonstrate that the lysate of the amoeba *Willaertia magna* C2c Maky is able to elicit potato defenses, and direct fungicidal activity against *Phytophtora infestans* was observed. Proof of efficacy was first obtained in greenhouse, with up to 80% disease reduction, and confirmed in field trials. Formulated products provided up to 77% protection in field in the case of low infestation (28%) and up to 49% protection when the untreated plants were 100% destroyed. *Willaertia magna* C2c Maky was also able to significantly increase yield by up to 30% in field trials.

## 1. Introduction

The estimated total world production of potatoes in 2020 was 359 million tons, with China being the largest producer, accounting for 18% of worldwide production [1]. Potato production in 2020 totaled 54 million tons in the European Union (EU) [1] and 21.3 million tons in the USA, with a value of all potatoes sold of USD 3.65 billion [2].

Late blight, caused by *Phytophtora infestans*, was responsible for the famine in Ireland at the end of the 19th century and is still a worldwide threat, not only to potato but also to tomato, causing an estimated USD 6.7 billion of yield losses every year owing to its capacity to rapidly destroy foliage and to infect tubers [3]. *P. infestans* is an oomycete-producing sporangia that is able to germinate in free water via a germ tube at 20–25 °C or by releasing wall-less zoospores at 10–15 °C [4]. Sexual reproduction of this oomycete, resulting in the formation of oospores, is the most important factor involved in survival of the fungus in the field and in increases in genotype diversity. The asexual reproductive cycle consists of spore germination, infection of leaves, sporulation, spore dispersal, and survival. Several infestation cycles can occur within 2 weeks and cause the destruction of entire fields. *P. infestans* germination occurs in particular under conditions oft high relative humidity and in low temperature areas. To avoid infection of the foliage, the use of fungicides and integrated pest management are mandatory [5]. Efficient fungicidal substances, such as metalaxyl or its stereo isomer metalaxyl-M (also known as mefenoxam), were formerly able to fight potato late blight (PLB); however resistance rapidly appeared, and their efficacy considerably declined [4]. Fluazinam has a broad spectrum of activity thanks to the interruption of the pathogen cell’s energy production. It has been used for more than 30 years in numerous commercial solutions against PLB (e.g., Banjo Extra^®^ by Adama France SAS, Vendetta^®^ by Cheminova, and Voyager^®^ by Belchim Crop Protection). It was considered to have a low resistance risk, but *P. infestans* genotype EU_33_A2, which was isolated in 2009 in the Netherlands and found in numerous location since then, is resistant to fluazinam [6]. This ability to resist fungicides may be due to the genetic flexibility of *P. infestans* linked to the presence of numerous transposons in its genome [7]. In the Netherlands, in some years, as many as 15 treatments are necessary to prevent the crop from infection by the pathogen (B. Evenhuis, Wageningen University, personal communication).

As mentioned above, the use of efficient crop protection practices is required. Improvement in the efficacy of active ingredients to decrease the number of treatments can reduce the environmental burden and achieve better results on terms of controlling late blight in the field. With the goal of sustainable agriculture, EU environmental plans call for a reduction in the use of chemical pesticides in crop treatments and for such pesticides to be replaced by natural substances [8]. A systematic map protocol is currently under construction to support the development of optimized methods for integrated pest management and to identify alternatives for potato disease control [9]. In 2019, the European Commission renewed copper authorization for 7 years and reduced its permissible use to an average of 4 kg per hectare per year. By 2026, the use of copper might be forbidden in all European countries, as is currently the case in Denmark and the Netherlands. In addition, mancozeb, a key standard fungicide used to fight PLB, was banned in 2021 in the EU [10]. Political and societal pressures foster the development of biological alternatives for organic production and for conventional agriculture.

In this context, Amoéba, an innovative French company, has developed a natural active substance to control plant diseases: the lysate of the amoeba *Willaertia magna* C2c Maky (C2c). Its efficacy is based on a dual mode of action (elicitation of plant defenses and anti-germinative properties) and was initially demonstrated on grape downy mildew [11]. Many conventional fungicides that are effective against *Plasmopara viticola* (responsible for grape downy mildew) can also control *P. infestans*. This is the case of mancozeb and mefenoxam, for instance. Copper is also effective on both diseases, although very few biofungicides are available against PLB. Some elicitors, such as cerevisane (ROMEO^®^, Agrauxine, Beaucouzé, France), and COS-OGA (FYTOSAVE^®^, Fytofend, Gembloux, Belgium and Syngenta, Basel, Switzerland), are, to the best of our knowledge, registered and used on grapes but not on potato.

We hypothesized that our active substance might also have an effect against other diseases caused by oomycetes in crops other than grapevine. In this paper, we present data obtained against PLB in greenhouse and in field trials with the raw dehydrated active substance (AS) AXP10 and four formulations: AXP08, AXP12, AXP13, and AXP15.

## 2. Results

### 2.1. Elicitor Effect on Potato Genes

The effect of C2c lysate on the expression of potato defense genes was evaluated under controlled conditions with the qPFD^®^ system based on 28 marker genes [12]. These genes represent the main defense pathways in plants, including those involved in parietal modification or signaling pathways (salicylic acid, jasmonic acid, and ethylene) and genes encoding the pathogenesis-related (PR) proteins.

In this experiment, there was no spread of pathogen or hydrogen peroxide (which can mimic a pathogen attack), so the observed effect is the direct effect of the product on plant defenses.

**Figure 1:**
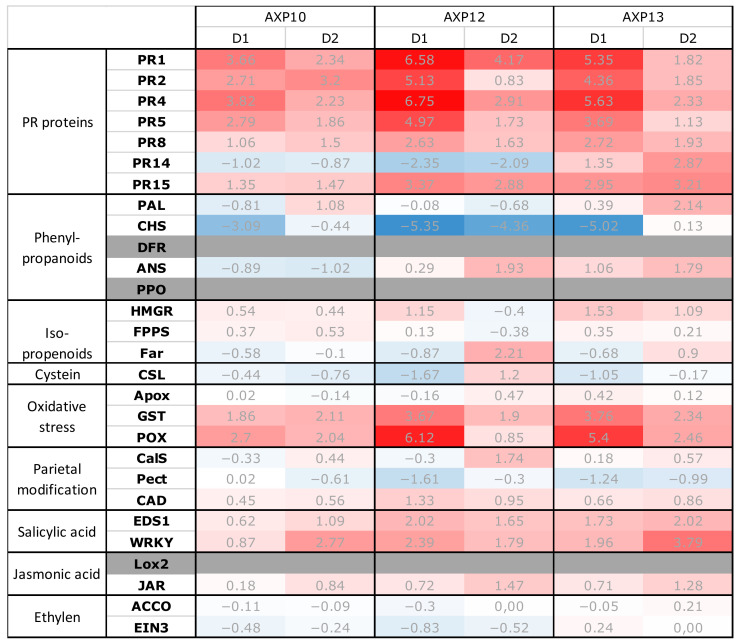
Heat map of the relative expressions of the 28 qPFD^®^ potato defense genes of the samples. Data are presented as the means of the biological replicates (R1, R2, and R3) calibrated by their respective H_2_O negative controls. Fold change represents relative gene expression values (2-ΔΔCt) of the defense genes (qPFD^®^) in potato, compared to the water control. Values superior or equal to 2 are indicated in light red (moderate inductions), and the highest values are in intense red (high inductions). Assessment of the three candidate products one (D1) and two (D2) days after treatment. Values inferior or equal to −2 are in indicated light blue (moderate repression), and the lowest values are indicated in intense blue (high repression). The average relative expressions were obtained by RT-qPCR from two independent repetitions and relative to the water control at each sampling date.

The DFR, PPO, and Lox2 encoding genes showed late amplifications or did not amplify, rendering these data unexploitable (shaded boxes in Figure 1) and were therefore not considered for further analyses. The defense gene expression level of the samples was normalized according to the reference genes encoding actin and glyceraldehyde-3-phosphate dehydrogenase (GAPDH).

The three tested products, AXP10 (raw dehydrated AS), AXP12 (SC formulation), and AXP13 (OD formulation), generated an overexpression of potato defense genes, which was higher one day after the last treatment (D1) compared to two days after the last application (D2), with D0 representing the day of the second application. The most noticeable responses were observed with AXP12, followed by AXP13 and AXP10. The overexpressed genes were globally similar from one modality to another, inducing PR, glutathione S-transferase (GST), and peroxidase (POX), as well as occasionally, WRKY transcription factor, enhanced disease susceptibility (EDS1), and fatty acyl-CoA reductase (Far) genes. The chalcone synthase (CHS) encoding gene was repressed for the different modalities, mainly at D1. Figure 1 shows that AXP10 promoted the overexpression of genes coding the PR1 to PR5 proteins and POX at D1 and the overexpression of genes coding the PR1 to PR4 proteins, GST, POX, and WRKY at D2. AXP12 elicited the defense genes coding the PR1 to PR8 and PR15 proteins, GST, POX, and EDS1 at D1. At D2, it promoted the overexpression of genes coding the PR1, PR4, and PR15 proteins, as well as Far. AXP13 elicited the defense genes coding the PR1 to PR8 and PR15 proteins, GST, and POX at D1. At D2, it promoted the overexpression of genes coding the PR4, PR14, and PR15 proteins, as well as PAL, GST, POX, EDS1, and WRKY. A difference in elicitation was observed between the AXP12 and AXP13 treatments with respect to the PR14 gene, which was repressed after application of the AXP12 formulation but overexpressed after application of the AXP13 formulation.

### 2.2. Greenhouse Efficacy Tests

#### 2.2.1. 2019 Greenhouse Trial

The objective of this greenhouse trial was to compare the efficacy of the raw dehydrated active substance (AXP10) at various concentrations (1.5, 4.5, and 7.5 g/L) and four formulations at an AXP10 concentration of 4.5 g/L (F1 to F4) versus *P. infestans* causing PLB.

**Figure 2:**
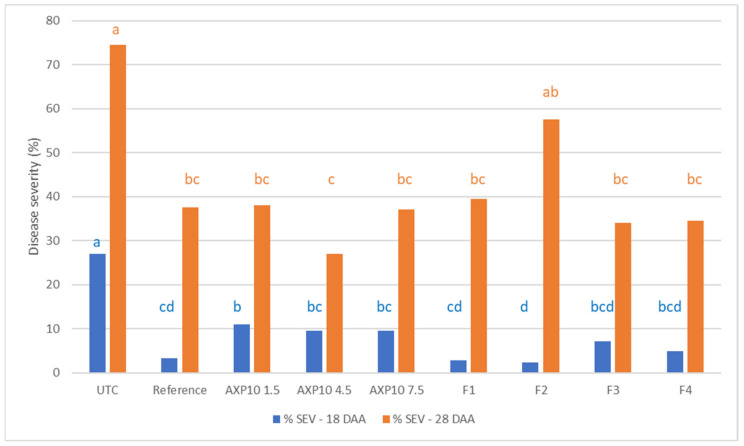

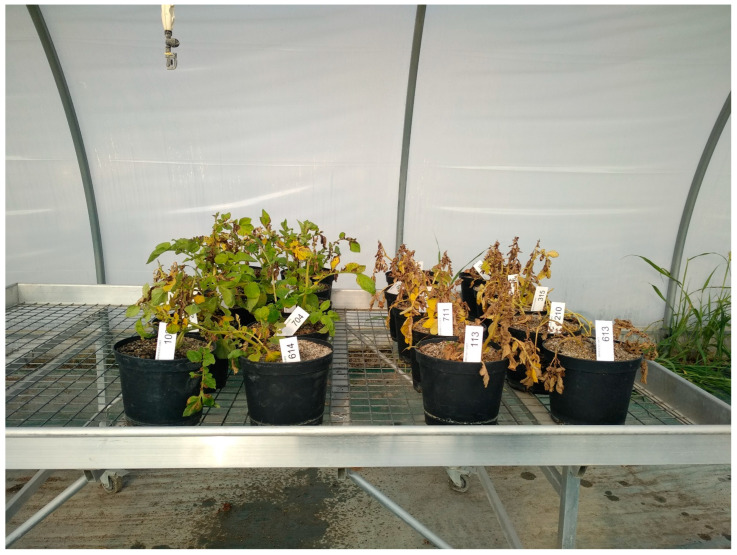
2019 greenhouse test. Top: disease severity evaluated on plant leaves with AXP10 at concentrations of 1, 4.5, and 7.5 g/L, four formulations (F1 to F4, all containing 4.5 g/L AXP10); and Dithane^®^ Neotec (3.2 g/L mancozeb) as the reference product; letters a, b, c and d; the same characters indicate that data are not significantly different (*p* = 0.05). The untreated control (UTC) was spread with demineralized water. Blue: notation of the disease severity 18 days after treatment application (18 DAA); orange: notation of the disease severity 28 days after treatment application (28 DAA). Bottom: photo of potato plants at the end of the experiment; left: potatoes treated with 4.5 g/L AXP10; right: UTC potatoes.

In the first assessment, 18 days after product application (18 DAA), the disease severity of the untreated control (UTC) was 27% on potato leaves, and the chemical reference Dithane^®^ (Neotec Indofil Industries, the Netherlands), applied at a concentration of 3.2 g/L of mancozeb), reduced the infection level to 3.3%. Except with AXP10 at a concentration of 1.5 g/L (11% of disease severity), the disease level remained below 10%, with an infection level ranging from 2.4% to 9.5% (Figure 2 Top). All treatments differed significantly from the UTC. The best protection was obtained with formulation F2, showing 91.1% efficacy, which was slightly better but not significantly different from the chemical reference, which provided 87.8% control against PLB. No phytotoxicity was observed.

Ten days later (28 DAA), the UTC disease severity reached 74.5%; all plants were infected (100% incidence), and a severity of 37.5% was observed with the chemical reference. With the exception of F2 (57.5% disease severity), the disease level remained below 40%, with an infection level ranging from 27% to 39.5% (Figure 2, Top). All treatments, except formulation F2, differed significantly from the UTC. The best protection (61.8% efficacy) was obtained with 4.5 g/L AXP10 (Figure 2, Bottom), which was slightly better but not significantly different from the chemical reference, which provided 37.5% protection to the plant. Formulation F2 provided the worst protection but did not significantly differ from UTC, with 32.3% efficacy.

#### 2.2.2. 2021 Greenhouse Trial

In this experiment, the efficacy of two formulations, AXP12 (SC formulation) and AXP13 (OD formulation), sprayed at different dose rates (0.625, 1.25, and 2.5 kg/ha) and time intervals (seven, three, one, and three plus one days before pathogenic infection) against *P. infestans* was tested in a pot experiment in comparison with Cuprozin^®^ progress containing copper hydroxide (Cu) at a concentration of 383 g/L. The aim of the experiment was to mimic agricultural practices as close as possible within an experimental setup.

**Figure 3:**
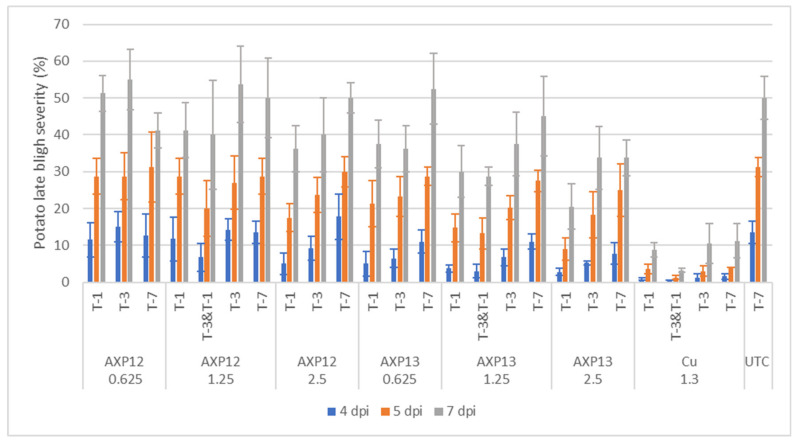
2021 greenhouse test: late blight severity at 4 (blue bars), 5 (orange bars), and 7 (grey bars) days post inoculation (dpi) on the whole plant. Spray timing: seven, three, or one day(s) before infection (T-7, T-3, and T-1, respectively) or three and one day(s) before infection (T-3 & T-1); AXP12 and AXP13 were sprayed at three rates: 0.625, 1.25, and 2.5 kg/ha; Cuprozin^®^ progress at 1.3 L/ha. Results are presented as the mean +/− standard deviation (*n* = 4).

No phytotoxicity was observed on the potato plants after spray application of the fungicides. The disease severity in the UTC increased from 14% 4 days post infection (4 dpi) to 50% 7 dpi (Figure 3). The reference product (Cu) performed well under all conditions, with a mean efficacy of 89%.

For statistical analysis, the data were log-transformed to meet the requirements for a normal distribution (Table 1). AXP12 at the lowest dose (0.625 kg/ha) did not significantly differ from the UTC. At the medium dose (1.25 kg/ha), the double treatment, with one and three -day(s) before infection treatment, significantly differed from the UTC at 4 and 5 dpi, with an efficacy of 50% and 36%, respectively. The highest dose of AXP12 (2.5 kg/ha) differed significantly from the UTC only when the treatment was applied one day before infection, with a decreasing efficacy from 63% at 4 dpi to 28% at 7 dpi. AXP13 at the lowest dose differed significantly from the UTC when applied one day before infection at 4 and 5 dpi with 63% and 32% efficacy, respectively and when applied three days before infection at 4 dpi with an efficacy of 52%. At the medium dose, AXP13 differed significantly from the UTC, except when the treatment was applied seven days before infection. At the highest dose, all conditions differed significantly from the UTC. The maximal efficacy of AXP13 was 80% for the high dose applied one day before infection at 4 dpi (Figure 3 and Table 1).

### 2.3. Field Trials

Two field trial campaigns were conducted in 2020 and 2021. The first type of trial was conducted to study the dose response of the formulated products by comparing their protection efficacy with respect to the reduction in the average disease severity in the treated plants compared to the UTV. Three product doses of AXP08 and AXP12 were tested with 250, 500, and 750, or 1000 g of active ingredient (gai)/ha in 2020, and two doses of AXP12 and AXP13 were tested at 125 and 250 gai/ha in 2021.

The area under the disease progress curve (AUDPC) is a useful quantitative summary of disease intensity over time for comparison across years, locations, or management tactics. A high AUDPC is synonymous with a high level of disease. It is used to compare the efficacy of the formulations in field trials.

Considering the highly susceptible potato variety Bintje, the results did not meet the expected efficacy in 2020 in trials 2020PTMDF, 20A6-02FP-1, and RFR20-133-518FE because of a high disease level that reached 60%, 100%, and 92%, respectively. However, in 2021, in the FR21F07 trial, a significant decrease in the disease was observed, with 48% efficacy when the severity in the UTC was 29% (Table 2). On less sensitive varieties, such as Ranger, Umatilla, Afra, Laura, Caesar, Challenger, El Mundo, Désirée, Eurogrande, and Euroflora, a mean efficacy of 56% was measured (Table 2).

**Figure 4:**
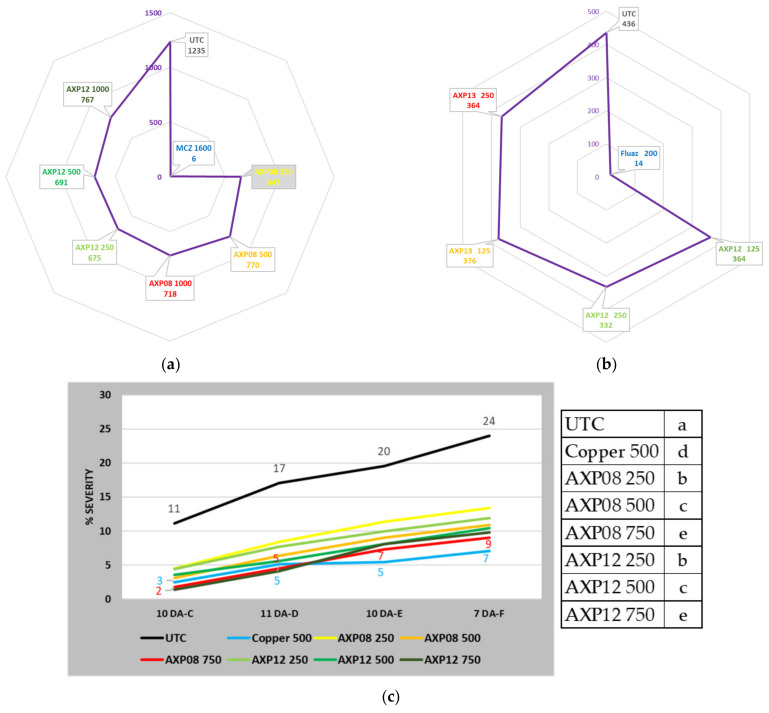

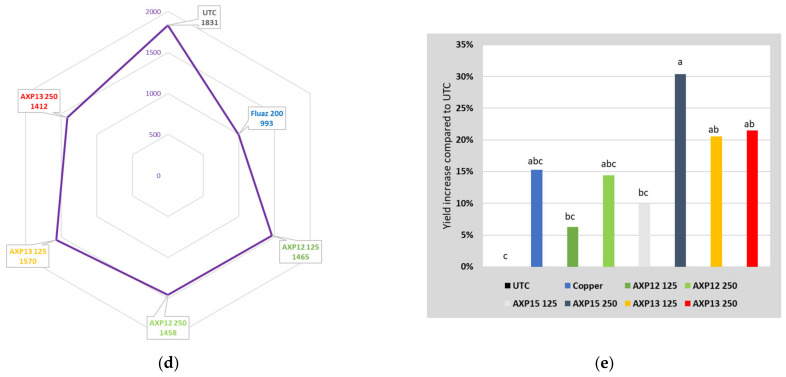
Field trial data on formulation efficacy: (**a**) AUDPC of the 20-42491-FR01 trial; (**b**) AUDPC of the 48677FR02 trial; (**c**) disease severity of field trial 225.F.SAG20 conducted in Italy; (**d**) AUDPC of the 48677BE03 trial; (**e**) yield increase in 2021PTMDF01 trial; letters; the same characters indicate that data are not significantly different (*p* = 0.05).

The 20-42491-FR01 trial, located in Inchy en Artois (France), was set up to evaluate the efficacy of AXP08 and AXP12 against PLB. The pressure regularly increased in the UTC: 1% of foliage destruction on 03/07/2020 (one day after treatment F: 1 DA-F) to 73% on 17/07/2020 (0 DA-I). Until the end of the trial, the pressure did not evolve: 78% on 20/07/2020 (2 DA-J) and 78% on 27/07/2020 (5 DA-J—last assessment). During the trial, Dithane^®^ Neotec at 2 kg/ha perfectly controlled the disease. At the end of the trial, only 0.7% of the foliage was destroyed. At the beginning of infestation (09/07/2020), at the peak of the disease (20/07/2020), and at the end of the trial (27/07/2020), AXP08 and AXP12 numerically reduced the intensity of the attack compared to the untreated plots; however, due to field variability, the disease decrease was not statistically significant. However, considering the whole trial with AUDPC, all conditions differed significantly from the UTC (Figure 4a). The best protection throughout the trial was obtained with AXP08 at 250 gai/ha (Table 2). No dose effect was observed.

The aim of the F536421 trial, conducted in Sours (France), was to compare the efficacy of AXP12 and AXP13 on PLB. Products AXP12 and AXP13 exhibited a significant fungicidal activity against PLB, with 58% efficacy of AXP12 at a dose of 250 gai/ha with a level of infestation of 38% (Table 2). The efficacy average was 40% to 60%, reaching 70% at the beginning of the infestation. At the end of the trial, AXP12 still exhibited fungicidal activity, with 15% efficacy, although this activity was not statistically significant.

The 21-48677-FR02 trial, conducted in Inchy en Artois (France), was set up to evaluate the efficacy of AXP12 and AXP13 against PLB. The first spots of the disease were observed on 28 June 2021 in the untreated plots. The second part of June and the first half of July were favorable for disease development, with regular rainfalls and mild temperatures (~15 °C during the night and 20–25 °C during the day). After the last application, regardless of the treatment, all plants were attacked. The development of the disease was fast in the untreated plots, i.e., 14 days after appearance, 86% of the foliage was destroyed (trial stopped on 12/07/2021). Except for the reference, which provided very high efficacy, control of the disease was difficult for both AXP formulations. The pressure in the environment and the climatic conditions were favorable to the disease. AXP12 was slightly more efficient than AXP13. APX12 showed a small dose effect, with 24% efficacy at the highest dose, differing significantly from the UTC (Table 2, Figure 4b).

The 225.F.SAG20 trial was set up in Luco dei Marsi (Italy) in a typical potato cultivation area. The efficacy of AXP08 and AXP12 at doses of 250, 500, and 750 gai/ha against PLB and that of the reference, KING containing tribasic copper sulfate (Diachem, Italy) at a dose of 500 gai/ha, were evaluated. In the presence of medium to severe PLB pressure, all tested products resulted in a significant reduction in disease symptoms in comparison to the UTC (Figure 4c). The best protection was provided by AXP08 750, with a global efficacy of 63% at the end of the trial (Table 2). Although AXP08 and AXP12 demonstrated significant performances, KING recorded the best overall results. A consistent dose response was observed with all tested dose rates of AXP08 and AXP12 (Figure 4c).

The 21-48677-BE03 trial, located in Perwez (Belgium), was set up to evaluate the efficacy of AXP12 and AXP13 at doses of 125 and 250 gai/ha against PLB. The first spots of disease were observed on 9 July 2021 in the untreated plots. Until mid-June, the climate was not favorable for disease development (dry conditions). After this date, the climate changed, and regular and heavy rainfalls were observed for one month, with more than 230 mm of rainfall recorded. The temperatures were then favorable for disease development, and 95% of the UTC was destroyed by the end of the trial (Table 2). Four days after the seventh application, with 79% disease severity in the UTC, AXP13 at a dose of 250 gai/ha reduced the disease with 52% efficacy (Table 2). Moreover, both AXP formulations, regardless of the rate, significantly reduced the disease, with a global efficacy of 23% (Figure 4d).

The purpose of the CT21-4-30DE1 trial, located in Uelzen-Molzen (Germany), was to supply data on the efficacy of AXP12, AXP13, and AXP15 applied at two rates 125 and 250 gai/ha. The first symptoms of infection occurred in all treatments on leaves seven days after the E treatment (7 DA-E). Infection increased up 61% by the end of the trial (Table 2). The maximal efficacy was obtained with AXP15 at a dose of 250 gai/ha (Table 2), with a disease reduction of 57% when the severity in the UTC was 35%. The potato tubers of this trial were harvested, and 100 healthy tubers were stored for four weeks. After storage, no rotten tubers were found in any of the treatments. No significant difference in yield was observed between the UTC and the treatments.

The 2021PTMDF01 trial, located in Vreden-Ammeloe (Germany), was conducted to assess the efficacy of AXP12, AXP13, and AXP15 at two doses (125 and 250 gai/ha) against PLB both on leaves and tubers under natural infestation conditions. The first application was conducted on 22 June 2021. The applications were conducted at intervals of 7 (±1) days. The last application was conducted on 25 August 2021 (a total of 10 treatments). First lesions with the disease were detected on 5 July in all treatments. In the UTC, the disease increased on leaves from 10.0% on 12 July to 100% on 25 August (Table 2). Compared to the UTC, significantly less infestation was observed for all treatments until 18 August, except for AXP12 at a dose of 125 gai/ha on 3 and 11 August and for AXP 13 at a dose of 125 gai/ha on 5 and 12 July. AXP15 at a dose of 250 gai/ha was the most efficient treatment, with 49% efficacy (Table 2), not differing significantly from the reference (51% efficacy). The harvest took place on 22 September. The yield ranged from 26.8 t/ha in the UTC to 34.9 t/ha with AXP 15 at a dose of 250 gai/ha, corresponding to a 30% yield increase (Figure 4e).

**Figure 5:**
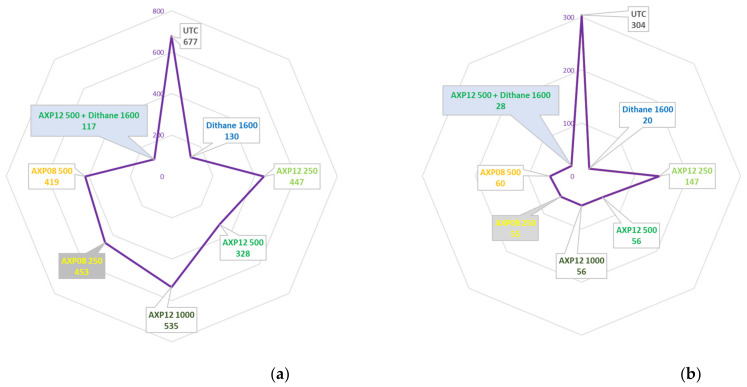

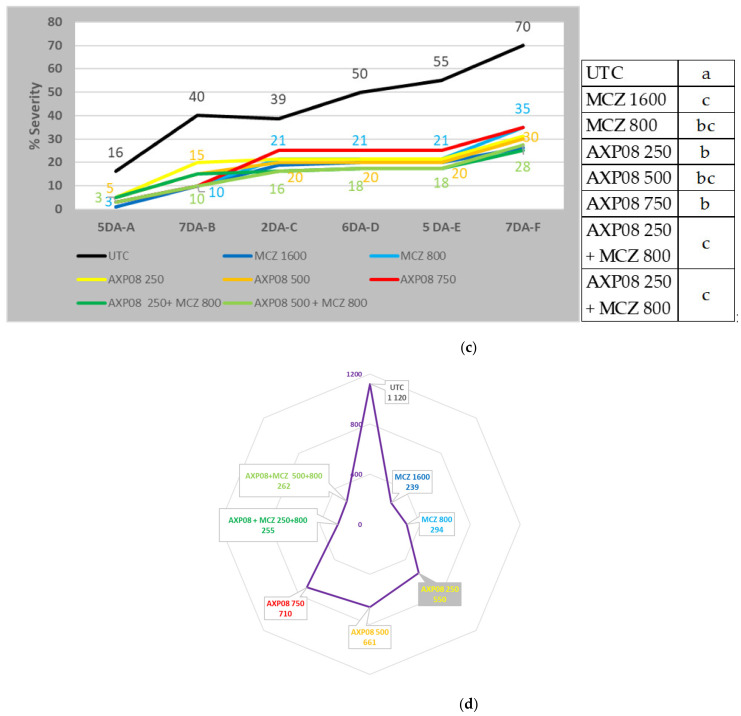
Field trial data on the dose rate and mancozeb complement: (**a**) AUDPC of the AmoebaLB2020 trial; (**b**) AUDPC of the AmoebaRR2020 trial; (**c**) disease severity of field trial CT20-4-95DE1; letters; the same characters indicated that data are not significantly different (*p* = 0.05) (**d**) AUDPC of trial SBN-20-42491-BE03.

Trials AmoebaLB2020 and RR2020 conducted in Bonners Ferry (USA) were designed to compare the efficacy of AXP08 at two doses (250 and 500 gai/ha) and AXP12 at three doses alone (250, 500, and 1000 gai/ha) and at one dose (500 gai/ha) in a tank mix with Dithane^®^ Neotec containing 800 gai/ha mancozeb. At the end of the trial, the disease severity in the UTC was 60% and 28% on leaves in the UTC in trials LB2020 and RR2020, respectively (Table 2). Data analysis revealed a reduction in the overall disease severity of 52% and 82% with AXP12 at a dose of 500 gai/ha and AXP08 at a dose of 250 gai/ha, respectively (Figure 5a,b). The best efficacy among unmixed doses was obtained with AXP12 at a dose of 500 gai/ha in the LB2020 trial (Table 2). In the RR2020 trial, AXP08 alone at a dose of 500 gai/ha was the best performer, not differing significantly from AXP12 at doses of 500 and 1000 gai/ha or AXP08 at the dose of 250 gai/ha (Figure 5b). The complementation test of AXP12 with Dithane^®^ Neotec showed an increase in efficacy from 81% to 83% in the LB2020 trial, although this result did not differ significantly from Dithane^®^ Neotec alone (Figure 5a). No complementation was observed in the RR2020 trial (Figure 5b). Potatoes were harvested in both trials. For LB2020, 58.5 T/ha were obtained for the UTC, and 65.2 T/ha were obtained with 250 gai/ha AXP08. However, this 11% increase was not statistically significant. Dithane^®^ Neotec provided a significant yield increase of 19%. No significant yield increase was obtained for any of the conditions in the RR 2020 trial, but 15%, 16%, and 19% yield increases were observed with Dithane^®^ Neotec, AXP08 at a dose of 250 gai/ha, and AXP12 at a dose of 500 gai/ha, respectively.

The CT20-4-95DE1 trial was conducted in Heidelberg (Germany). The purpose of the study was to supply data on the efficacy of AXP08 against PLB applied at three rates alone (250, 500, and 750 gai/ha) and at two rates (250 and 500 gai/ha) in a tank mix with Dithane^®^ Neotec at a rate of 800 gai/ha mancozeb. Six treatments were performed (A to F) with 5- to 7-day intervals, depending on the disease pressure. Symptoms of *P. infestans* were visible on leaves with 1% severity in all plots at the time of the first application and increased rapidly in the untreated plots to 70% at the time of last assessment 7 days after the last treatment (7 DA-F). Significant differences were observed in the treated conditions. The best efficacy was obtained with AXP08 at a dose of 500 gai/ha. The same efficacy was obtained with Dithane^®^ Neotec at a dose of 1600 gai/ha alone and at a dose of 800 gai/ha mixed with AXP08 at doses of 250 and 500 gai/ha (Figure 5c). Potatoes were harvested. All treatments resulted in significant higher yield in comparison to the UTC. The best results were obtained with Dithane^®^ Neotec at a dose of 1600 gai/ha and with AXP08 at a dose of 500 gai/ha mixed with Dithane^®^ Neotec at a dose of 800 gai/ha, with a production of 21.2 and 20.2 T/ha, respectively. A significant decrease of 74% of rotten tubers was observed in potatoes treated with AXP08 at a dose of 500 gai/ha mixed with Dithane^®^ Neotec at a dose of 800 gai/ha.

**Figure 6:**
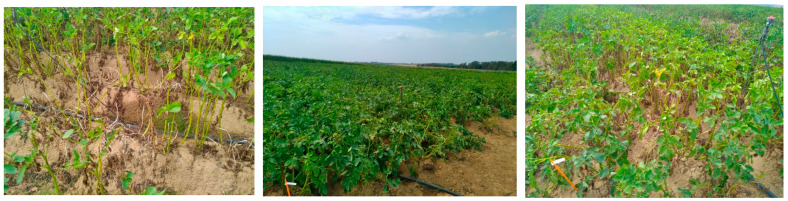
Photos from field trial SBN-20-42491-BE03. Left: UTC; middle: mancozeb 1600 gai/ha; right: AXP08 500 gai/ha.

The 20-42491-BE03 trial, located in Malèves-Sainte-Marie-Wastine (Belgium), was set up to evaluate the selectivity and efficacy of AXP08 alone or in a tank mix with Dithane^®^ Neotec against PLB (Figure 6). On 10/08/2020 (4 DA-F), AXP08 at a dose of 250 gai/ha provided up to 58% efficacy (Table 2). With regard to AUDPC, AXP08 at a dose of 250 gai/ha was numerically more efficient than the other two rates (Figure 5d). In association with Dithane^®^ Neotec at a dose of 800 gai/ha, no effect of AXP08 was observed compared to Dithane^®^ Neotec alone (Figure 5d). On 19/08/2020 (6 DA-G), a slight decrease in efficacy was observed for AXP08 a dose of 250 gai/ha, providing 50% efficacy. On 31/08/2020 (11 DA-H), no persistence of action was observed for AXP08 (an efficacy of 5–10%). However, in terms of overall protection (AUDPC), AXP08 at a dose of 250 gai/ha was significantly more efficient than the 750 gai/ha rate. Applied at a rate of 500 gai/ha, the efficacy was intermediate (Figure 5d).

## 3. Discussion

PLB is difficult to control and can sometimes be explosive in sensitive potato varieties such as Bintje. The efficacy of fungicides in controlling PLB depends on their characteristics, the dose rate, and the time between the spray application and infection by *P. infestans*. Chemical fungicides, such as Shirlan^®^ (ISK Biosciences Europe S.A., Diegem, Belgium), Infinito (Bayer Crop Science, Leverkusen, Germany), and Acrobat^®^ (BASF, Ludwigshafen, Germany), have the capacity to inhibit the development and spread of *P. infestans*, but resistances are increasing in frequency. For example, strains resistant to phenylamide fungicides (e.g., metalaxyl, furalaxyl, or ofurace) occur widely in Europe [5]. The *P. infestans* genome is quite large (~240 Mb) and full of transposons [4,7,13]. This genetic particularity could explain its ability to resist conventional fungicides. Even mancozeb, which has been used for decades, is not always able to control PLB. Moreover, this pesticide was banned last year in the EU. Copper and its salts are the only substances that can currently be used in the organic production of potato, with a medium efficacy against PLB [14]. However, due limitations on the use of copper to a maximum of at 4 kg/ha/year and a possible ban in 2026 in the EU, alternatives are needed.

The aim of this study was to evaluate the ability of a lysate of the amoeba *W. magna* C2c Maky, manufactured by Amoéba (Chassieu, France), to fight PLB. C2c activity against plant pathogens was previously demonstrated on grape downy mildew. Its efficacy was explained by a conjunction of plant defense gene stimulation and antifungal activity [11]. The same approach was used against PLB, and the activation of potato defense genes was demonstrated by the raw substance AXP10 and by the two formulations AXP12 and AXP13 (Figure 1). The most enhanced genes are those encoding PR1 to 8, PR15, GST, POX, and WRKY. A pathogenic attack induces the activation of multiple defense mechanisms, both passive and active. The active method requires de novo protein synthesis, which is regulated by a complex network involving three main molecules: salicylic acid, jasmonic acid, and ethylene. Products AXP10, −12, and −13 were able to stimulate WRKY expression involved in the salicylic pathway. The WRKY transcription factors play an important role in plant growth, development, and defense responses [15,16], and their stimulation can help plants to resist pathogen attacks. The consequence of these gene activations is an increase in the synthesis of PR proteins, as observed in the present study (Figure 1). In parallel, the stimulation of plant defense genes induces oxidative stress. The class III plant POX is well-known to participate in the metabolism of reactive oxygen species and to be involved in host cell death at the infection site, limiting pathogen progression [17]. POX synthesis was induced by AXP10, −12, and −13 (Figure 1). The only significantly repressed gene codes the CHS, from the family of polyketide synthase enzymes that is usually associated with plant defense mechanisms [18]. This inhibition could be due to the presence of flavonoid analogs in AXP10, as CHS can be inhibited by naringenin [19].

The efficacy of AXP10 and formulations against PLB was first demonstrated in 2019 through a greenhouse experiment (Figure 2) and confirmed in 2021 with AXP12 and AXP13 products. The 2021 greenhouse experiment involved inoculation with a *P. infestans* isolate belonging to the clonal lineage EU-36-A2. In northwest Europe, EU36-A2 is the dominant genotype of the *P. infestans* population. EU-36-A2 is generally accepted as an aggressive type of *P. infestans* on potato. The efficacy of Cuprozin^®^ progress in controlling PLB was approximately 82% to 95%, depending on the interval between spray application and inoculation, achieving effective control of the disease. AXP12 and AXP13 were able to control the disease with a maximal efficacy of 63% and 80%, respectively. These data demonstrate that the OD formulation could be slightly more adapted to potato protection than the SC formulation, probably because lipophilic surfactants help molecules to penetrate the plant cuticle [20]. For both formulations, a tendency for a dose response effect was observed, and this effect was found to be most prominent when AXP12 or AXP13 was sprayed one day before pathogen inoculation.

Moving from the greenhouse to the field is not always easy, and products demonstrating a high efficacy under controlled conditions can be inefficient in the field because of harsh environmental conditions (rain, UV, pathogen aggressivity, etc.). Field trials conducted in 2020 and 2021 demonstrated that AXP12 and AXP13 were efficient against PLB in most cases (Table 2). Highly sensitive varieties, such as Bintje, remain a challenge in fighting PLB for organic production. The severity of the disease is also an important parameter. With a severity of 28%, the level of protection reached 77%, and with a severity of 100%, the protection decreased to 49% (Table 2), although the yield was significantly increased by 30% (Figure 4e). Other natural compounds originating from microorganisms are known to be able to fight against PLB. For example, volatile organic molecules excreted by bacterial endophytes in potatoes inhibit the mycelial growth of *P. infestans* by up to 49% and suppress sporangia germination, both in vitro and on detached potato leaflets [21]. In tubers, biocontrol of PLB was achieved from a collection of *Pseudomonas* spp. strains producing phenazine-1-carboxylic acid. [22]. Metabolites extracted from *Myxococcus* strains demonstrated activity against PLB [23,24]. Fungicidal activity was also observed with ethyl-acetate extracts from strains belonging to the fungal genus *Chaetomium* [25]. Living microorganisms, such as bacterial strains of *Streptomyces*, *Pseudomonas*, *Saccharothrix, Bacillus*, and *Nocardiopsis*, are also able to act as biological control agents against PLB [26,27,28,29]. However, these molecules and strains have not been tested under field conditions.

Sustainability in agriculture in conjunction with disease fighting could be achieved by associating biological treatment with integrated field management. A northern European study demonstrated a correlation between crop rotation and incidence of PLB, in part because oospores in soils remain infectious for several years [5] and can survive in the form of mycelia in potato tubers [30]. Decision support systems such as PLANT-Plus (Dacom PLANT-Service B.V.), can also help to control PLB [31], although cultivar resistances require specific management [32]. Decision support systems are also useful to reduce the number of treatments [14]. Pandit et al. [33] summarized existing and incoming strategies to control plant pathogens using biological approaches, for example, the use of microbial strains and natural compounds to provide a natural protection. Algae (micro and macro) possess antibacterial, antiviral, antifungal, nematicide, insecticidal and herbicidal properties and represent a vast reservoir of unknown properties [34]. Amoebas are even less known and represent a considerable reservoir of unknown properties.

In conclusion, in this study, we developed, for the first time, a biocontrol product based on a microorganism that is able to fight late blight in potatoes. *W. magna* C2c Maky lysate has the rare property of a dual mode of action;it is a plant elicitor (indirect effect) and inhibits the development and spread of *P. infestans* (direct effect). This dual mechanism is a guarantee of reliability—a real asset. Moreover, it is a safe product for human health and the environment, whereas the only natural product currently on the market to fight PLB is copper, which is known for its toxicity and ecotoxicity. The products developed by Amoéba are easy to use thanks to their formulations, and they do not require farmers to change their habits. Finally, the efficacy of the amoeba lysate was proven to be significant under greenhouse conditions and, most importantly, in fields. It is important to point out that the difference in performance observed between highly susceptible varieties and partially tolerant varieties implies the need for a combination of genetic control and biocontrol. Biocontrol products are not intended to be used alone throughout the season. Further experiments will be conducted in a practical treatment program against PLB to replace at least one chemical or copper treatment when the disease pressure is not excessively high in order to limit residue accumulation in soils. In parallel, other plant pathogens will be targeted to evaluate the range of activity of the lysate of *W. magna* C2c Maky.

## 4. Materials and Methods

### 4.1. Biocontrol Products

The raw dehydrated active substance (AS) AXP10 and four formulations AXP08, AXP12, AXP13, and AXP15, were used in this study. AXP08 is a wettable powder containing 60.5% AS (*w*/*w*) that was formulated from liquid lysate and dried by a spray-drying process. AXP12 is an aqueous suspension concentrate (SC) containing 20% AS (*w*/*w*) that was prepared from AXP10. AXP13 is an oil-based suspension concentrate (OD) containing 21.7% AS (*w*/*w*) that was prepared from AXP10. AXP15 is an SC containing 21.2%AS developed for the American organic market.

### 4.2. Anti-Oomycetal Tests under Greenhouse Conditions

Two assays were performed, one in 2019 at Syntech Research (Nîmes, France) with an environmental isolate of *P. infestans* and one in 2021 at Wageningen University and Research (Lelystad, The Netherlands) on a *P. infestans* isolate belonging to the clonal lineage EU-36-A2.

#### 4.2.1. 2019 Greenhouse Trial

The trial was performed in accordance with good experimental practice (GEP).

The plant variety used for the tests was Elodie, which is known to be highly susceptible to *P. infestans*. One plant per pot was planted, with 10 pots per modality. The raw active substance AXP10 was diluted in water at 1.5, 4.5, and 7.5 g/L, and four wettable powder (WP) formulations (F1 to F4) were tested at 9 g/L, each containing 4.5 g/L AXP10. These were compared with a chemical reference consisting of Dithane^®^ Neotec at 2 kg/ha and an untreated modality. A single application was performed on 9 April 2019 as a preventive application in the crop BBCH 21–25. All treatments were applied as a foliar application at 400 L/ha using a backpack sprayer and a 1 m wide boom equipped with Teejet DG11002VS nozzles. Spraying was performed in a preventive manner, 24h before infection. An artificial inoculation was performed 24 h after treatment application.

Two efficacy assessments were performed on 27 April 2019 and 7 May 2019 considering the percentage area of the foliage affected by the disease (severity, PESSEV). A selectivity assessment (percentage of phytotoxicity, PHYGEN) was performed on 12 April 2019.

For each condition, a mean disease severity score was calculated by averaging the attack rates obtained for the 10 plants of the same condition. From this mean disease severity score, the efficacy (*E*) of the product for each condition could be calculated by comparison with the mean disease severity score obtained in the control condition (untreated plants) according to the following formula:

$$E=\frac{\left(T-D\right)}{T}\times 100$$

where *T* is the mean disease severity score obtained for the control condition (untreated plants), and *D* is that observed in the plants treated with tested fungicides at varying doses.

Statistical significance was determined through the use of analysis of variance (ANOVA) on untransformed data and on transformed data according to the Bartlett’s test.

#### 4.2.2. 2021 Greenhouse Trial

This study was conducted at the Wageningen Research Foundation business unit field crops.

Cultivated potato plants (cultivar Agria) were grown in pots to a height of 25–30 cm. The two amoeba products, AXP12 and AXP13, were sprayed at three doses in comparison with the reference, Cuprozin^®^ progress (Cu), and an untreated control (UTC), as listed in Table 3.

The potato plants were sprayed in a spraying cabin using a spray boom with three Airmix 110-05 spray nozzles at 2.5 bar, placed 50 cm apart, with a moving speed of 5 km/h approximately 40 cm over the top of the potato plants. The spray volume was 500 L/ha. Four plants per condition were treated. Spray applications were conducted on 22 April (T-7), 26 April (T-3), and 28 April (T-1) 2021. A *P. infestans* isolate belonging to the EU-36-A2 clonal lineage was used to inoculate plants on 29 April 2021. The inoculum density was set at about 5000 spores/mL. Inoculation was conducted by spraying potato plants with 10 mL of inoculum per plant.

Four notations of disease were performed. The percentage of necrotic foliage per plant was visually estimated. Average disease severity was calculated per assessment date. A phytotoxicity assessment was performed.

Analysis of variance on the parameters was performed using GENSTAT 19th edition.

### 4.3. Plant Defense Gene Study by qPFD^®^

#### 4.3.1. Biological Material

The trials were conducted under semi-controlled conditions in a greenhouse by Vegenov (Saint Pol de Léon, France) on potato plants of the Désirée variety. Potato cuttings were taken from apexes of the same leaf cluster. The plants were then grown in pots in potting soil.

#### 4.3.2. Plant Treatment and Samplings

At the 4th leaf stage, potato plant leaves were treated with a glass sprayer up to the runoff limit (~8 mL/plant). A total of 4 modalities were evaluated:

- Water (negative control);
- AXP10: AS not formulated (1 g/L);
- AXP12: formulation (1 or 5 g/L); and
- AXP13: formulation (1 or 5 g/L).

The trials were conducted following a three-block randomization design with three plants per modality for each sampling date (different plants were used for each sampling date).

**Figure 7:**
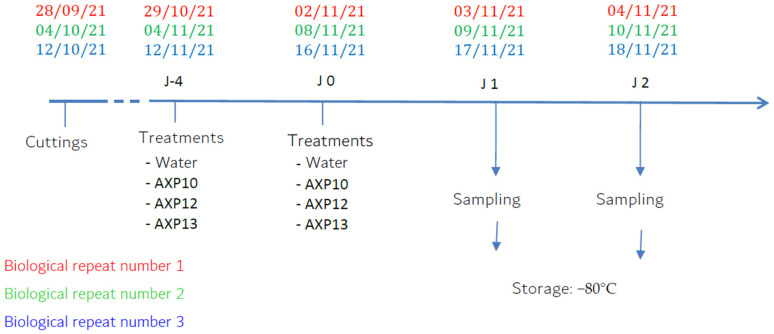
Experimental scheme of the three biological repeats.

Each modality was sprayed twice in a 4-day interval (J-4 and J0). Leaf samples from the third leaf stage were collected from three different plants (technical replicates) one (J1) and two (J2) days after the last spray and pooled in a single tube before analysis. The tubes were then immediately immersed in liquid nitrogen to limit RNA degradation and stored at −80 °C until extractions were performed (Figure 7).

#### 4.3.3. Gene Expression Monitoring

Gene expression analysis of the potato plantlets was performed using the quantitative RT-PCR microplate/DNA chip low density (qPFD^®^) method [12,35]. Briefly, RNA was extracted using a Macherey Nagel Nucleospin^®^ RNA plant and fungi kit, and DNase treatment was performed during extraction according to the supplier’s instructions.

RNA quantification and quality control were performed for each sample. The yield and quality of the extracted RNA were assessed with a spectrophotometer (Nanodrop ND-1000). For each RNA sample, a reverse transcription (RT) step was performed from RNA normalized to 200 ng/μL with an Invitrogen™ SuperScript™ II reverse transcriptase kit according to the supplier’s instructions. The expression levels of 28 defense genes (Table 4) were quantified in triplicate by RT-PCR (SYBR Green) with the qPFD^®^ tool [12]. Results were analyzed according to the 2-ΔΔCT method, which provides the relative expressions of defense genes in a given sample compared with a control sample (water), expressions normalized by the geometric mean of 3 reference genes (i.e., *tuA*, actin, and GAPDH) [12] from that sample and transformed into log 2 (gene expression profiles are visualized as a density map called a heat map). Overexpression and repression levels of the defense genes were considered significant above a threshold ≥2 or ≤2, respectively.

### 4.4. Field Trials

Fifteen field trials are reported herein. Nine were conducted in 2020 (seven in Europe and two in the USA) to assess the efficacy against PLB of two formulated products, AXP08 (WP) and AXP12 (SC). Two protocols were tested. The aim of the first protocol, 2020PTMDF, was to compare the two formulations at three doses, and the aim of the second protocol, 2020PTMDA, was to test the association of two rates of AXP08 with a reduced dose of the chemical reference, Dithane^®^ Neotec (Indofil Industries, Netherlands) compared to two rates of Dithane^®^ Neotec and three rates of AXP08 (Table 5).

Six field trials conducted in Europe in 2021 are presented to assess the efficacy against PLB of two (2021PTMDF1) or three (2021PTMDF2) formulated products, AXP12, AXP13 (OD), and AXP15 (SC) at two doses (Table 6).

The year, field trial reference, contractor, country, potato variety, and number of applications with number of days between two treatments are listed for each trial in Table 7.

The observations were focused on the severity of the disease on potato leaves and on the quantitative summary of disease intensity over time (AUDPC). In 5 cases (AmoebaLB2020, AmoebaRR2020, CT20-4-95DE1, CT21-4-30DE1, and 2021PTMDF01), tubercules were harvested, and the yield was determined (Table 2). The trial design, test compound efficacy evaluations, and phytotoxicity assessments were all performed in compliance with the principles of good experimental practices according to the European and Mediterranean Plant Protection Organization guidelines, which define the standard procedures for the evaluation of plant protection products. The application and evaluation dates were expressed according to the Biologische Bundesanstalt, Bundessortenamt and CHemical industry (BBCH) code 55, corresponding to specific developmental stages of the plant.

### 4.5. Statistical Analyses

Statistical significance was determined through the use of analysis of variance (ANOVA) (Kruskal–Wallis test and multiple pair-wise comparison Dunn test).

## Figures and Tables

**Table 1:** Log transformed severity (%) at 4, 5, and 7 -days post inoculation (dpi) of *P. infestans*. Green: significant differences in AXP12 and AXP13 compared to the UTC. Black: non-significant severity data followed by letters; the same characters indicated that data are not significantly different (*p* = 0.05).

Treatment kg/ha	Spray Time	4 dpi	5 dpi	7 dpi
**AXP12** **0.625**	**T-1**	10.9 ijk	28.5 jk	51.1 hijk
	**T-3**	14.6 k	28.2 ijk	54.6 k
	**T-7**	11.8 ijk	30.3 k	41.0 efghijk
**AXP12** **1.25**	**T-1**	10.8 ijk	28.5 jk	40.8 efghijk
	**T-3&T-1**	6.1 efg	19.0 efgh	38.1 defghi
	**T-3**	14.1 jk	26.3 hijk	53.0 jk
	**T-7**	13.3 jk	28.5 jk	49.0 fghijk
**AXP12** **2.5**	**T-1**	4.5 def	17.2 def	35.9 def
	**T-3**	8.9 ghij	23.4 fghijk	39.2 defghij
	**T-7**	16.9 k	29.8 k	49.9 ghijk
**AXP13** **0.625**	**T-1**	4.5 def	20.6 fghij	37.1 defgh
	**T-3**	6.2 efg	22.8 fghijk	35.9 def
	**T-7**	10.7 hijk	28.7 jk	51.9 ijk
**AXP13** **1.25**	**T-1**	3.7 de	14.4 de	29.5 d
	**T-3&T-1**	2.7 cd	12.7 d	28.7 d
	**T-3**	6.5 fgh	20.1 efghi	36.8 defg
	**T-7**	10.9 ijk	27.4 ijk	44.1 efijk
**AXP13** **2.5**	**T-1**	2.7 cd	8.6 c	19.7 c
	**T-3**	5.2 ef	17.4 defg	33.0 de
	**T-7**	7.5 fghi	24.4 ghijk	33.5 de
**Cu** **1.3**	**T-1**	0.9 ab	3.4 b	8.6 b
	**T-3&T-1**	0.2 a	1.2 a	3.2 a
	**T-3**	0.9 ab	2.8 b	9.6 b
	**T-7**	1.5 bc	4.0 b	10.7 b
**UTC**	**T-7**	13.3 jk	31.2 k	49.8 ghijk
**F pr.**		<0.001	<0.001	<0.001

**Table 2:** Best efficacy of products compared to the UTC. Y: yes, N: no; UTC: untreated control.

Trial Reference	Severity in UTC *	Product	Yield	AUDPC	Dose (gai/ha)	Best Product Efficacy/Severity in UTC
2020PTMDF	100%	None	N	N		0%/60%
20A6-02FP-1	100%	None	N	N		0%/100%
RFR20-133-518FE	92%	None	N	N		0%/92%
FR21F07	59%	AXP13	N	N	250	48%/29%
20-42491-FR01	78%	AXP08	N	Y	250	48%/78%
F536421	66%	AXP12	N	N	250	58%/38%
21-48677-FR02	86%	AXP12	N	Y	250	24%/86%
225.F.SAG20	24%	AXP08	N	N	750	63%/24%
21-48677-BE03	95%	AXP13	N	Y	250	52%/79%
CT21-4-30DE1	61%	AXP15	Y	N	250	57%/35%
2021PTMDF01	100%	AXP15	Y	Y	250	49%/100%
AmoebaLB2020	60%	AXP12	Y	Y	500	56%/40%
AmoebaRR 2020	28%	AXP08	Y	Y	250	77%/28%
CT20-4-95DE1	70%	AXP08	Y	Y	500	59%/70%
20-42491-BE03	84%	AXP08	N	Y	250	58%/30%

*: severity in UTC at the last assessment.

**Table 3:** Treatments and fungicides applied seven days (T-7), three days (T-3), one day (T-1), or both three and one day(s) (T-3&T-1) before *P. infestans* inoculation.

Fungicide	Active Ingredient	Dose Rate (L or kg/ha)	Day	Before	*P. infestans*	Inoculation
			T-7	T-3	T-1	T-3&T-1
UTC	-	-	A *	-	-	-
Cuprozin	Cu 383 g/L	1.3	B	I	T	K
AXP12	200 g/kg	0.625	C	L	U	-
AXP12	200 g/kg	1.25	D	M	W	N
AXP12	200 g/kg	2.5	E	O	X	-
AXP13	217 g/kg	0.625	F	P	Y	-
AXP13	217 g/kg	1.25	G	Q	Z	R
AXP13	217 g/kg	2.5	H	S	AA	-

* The letter code indicates the specification of the treatments.

**Table 4:** List of the studied plant defense genes [12].

Defense Classes and Subclasses		Defense Genes	
		Gene Code	Complete Name
Chemical and/or physical barriers	PR proteins	PR-1	Pathogenesis-related protein 1
		PR-2	Pathogenesis-related protein 2 (glucanases)
		PR-4	Pathogenesis-related protein 4 (hevein-like)
		PR-5	Pathogenesis-related protein 5 (thaumatin-like, osmotin)
		PR-8	Pathogenesis-related protein 8 (class III chitinase)
		PR-14	Pathogenesis-related protein 14 (lipid transfer protein)
		PR-15	Pathogenesis-related protein 15 (oxalate oxidase)
	Phenylpropanoids	PAL	Phenylalanine ammonia-lyase
		CHS	Chalcone synthase
		DFR	Dihydroflavonol reductase
		ANS	Anthocyanidin synthase
		PPO	Polyphenol oxidase
	Isoprenoids	HMGR	Hydroxymethyl glutarate-CoA reductase
		FPPS	Farnesyl pyrophosphate synthase
		Far	(E,E)-alpha-farnesene synthase
	Cysteines	CSL	Alliinase
	Oxidative stress	APOX	Ascorbate peroxidase
		GST	Glutathion S-transférase
		POX	Peroxidase
	Parietal modification	CalS	Callose synthase
		Pect	Pectin methyl esterase
		CAD	Cinnamyl alcohol dehydrogenase
Hormonal signaling	Salicylic acid (SA)	EDS1	Disease resistance protein EDS 1
		WRKY	WRKY transcription factor 30
	Jasmonic acid (JA)	LOX2	Lipoxygenase AtLOX2
		JAR	Jasmonate resistant 1
	Ethylene (ET)	ACCO	1-aminocyclopropene-1-carboxylate oxidase
		EIN3	EIN3-BINDING F BOX PROTEIN 1

**Table 5:** 2020 field protocols; CONC.: concentration, AS: active substance, UTC: untreated control.

2020PTMDF	Product	Form	CONC.	rate (kg/ha)	AS	Rate (g/ha)
1	UTC					
2	DITHANE	WP	80%	2.00	mancozeb	1600
3	AXP08	WP	60.5%	0.42	amoeba lysate	250
4	AXP08	WP	60.5%	0.84	amoeba lysate	500
5	AXP08	WP	60.5%	1.25	amoeba lysate	750
6	AXP12	SC	20%	1.25	amoeba lysate	250
7	AXP12	SC	20%	2.50	amoeba lysate	500
8	AXP12	SC	20%	3.75	amoeba lysate	750
**2020PTMDA**	**Product**	**Form**	**CONC.**	**Rate (kg/ha)**	**AS**	**Rate (g/ha)**
1	UTC					
2	DITHANE	WP	80%	2.00	mancozeb	1600
3	DITHANE	WP	80%	1.00	amoeba lysate	800
4	AXP08	WP	60.5%	0.42	amoeba lysate	250
5	AXP08	WP	60.5%	0.84	amoeba lysate	500
6	AXP08	WP	60.5%	1.25	amoeba lysate	750
7	AXP08 + DITHANE	WP		0.42 + 1	amoeba lysate	250 + 800
8	AXP08 + DITHANE	WP		0.84 + 1	amoeba lysate	500 + 800

**Table 6:** 2021 field protocols; CONC.: concentration, AS: active substance, UTC: untreated control, gai: grams of active substance.

2021PTMDF1	Product	Form	CONC.	Rate (kg/ha)	AS	Rate (gai/ha)
1	UTC					
2	Standard	SC	50/25%	0.40/2.5	Fluazinam/Copper	200/500
3	AXP12	SC	20%	0.63	amoeba lysate	125
4	AXP12	SC	20%	1.25	amoeba lysate	250
5	AXP13	OD	21.7%	0.63	amoeba lysate	125
6	AXP13	OD	21.7%	1.25	amoeba lysate	250
**2021PTMDF2**	**Product**	**Form**	**CONC.**	**Rate (kg/ha)**	**AS**	**Rate (gai/ha)**
1	UTC					
2	Standard	SC	25%	2.5	Copper	500
3	AXP12	SC	20%	0.63	amoeba lysate	125
4	AXP12	SC	20%	1.25	amoeba lysate	250
5	AXP15	SC	21.2%	0.63	amoeba lysate	125
6	AXP15	SC	21.2%	1.25	amoeba lysate	250
7	AXP13	OD	21.7%	1.25	amoeba lysate	250
8	AXP13	OD	21.7%	1.25	amoeba lysate	250

**Table 7:** Field trial characteristics; T: number of treatments, D: number of days between two treatments, UTC: untreated control; Ag: agricultural.

Year	Trial Reference	Contractor	Country	Variety	Applications
2020	AmoebaLB2020	Hubbard	USA	Umatilla	7T–7D
2020	AmoebaRR 2020	Hubbard	USA	Ranger Russet	7T–7D
2020	CT20-4-95DE1	Crop Trials	Germany	Afra	12T–5/7D
2020	2020PTMDF	Hetterich	Germany	Bintje	6T–7D
2020	225.F.SAG20	Sagea	Italy	Laura	6T–10/11D
2020	20-42491-FR01	Staphyt	France	Caesar	12T–7D
2020	20A6-02FP-1	SciencesAgro Atlantique	France	Bintje	6T–7D
2020	RFR20-133-518FE	Syntech	France	Bintje	12T–6/8D
2020	20-42491-BE03	Staphyt	Belgium	Challenger	8T–7D
2021	F536421	CentreExpé	France	El Mundo	10T–4/12D
2021	FR21F07	Ephydia	France	Bintje	6T–4/8D
2021	21-48677-FR02	Staphyt	France	Désirée	8T–3/7D
2021	21-48677-BE03	Staphyt	Belgium	Challenger	10T–4/6D
2021	CT21-4-30DE1	Crop Trials	Germany	Eurogrande	10T–7D
2021	2021PTMDF01	Hetterich	Germany	Euroflora	10T–6/8D

## Data Availability

The data presented in this study are available on request from the corresponding author. The data are not publicly available due to privacy.
